# Advances in human norovirus research: Vaccines, genotype distribution and antiviral strategies

**DOI:** 10.1016/j.virusres.2024.199486

**Published:** 2024-10-23

**Authors:** JunLi Chen, ZhengChao Cheng, Jing Chen, Lingling Qian, Haoran Wang, YuWei Liu

**Affiliations:** a Department of Laboratory Medicine, Department of Microbiology, School of Medicine, Jiangsu University, Zhenjiang, Jiangsu 212003,PR China; b Central laboratory of Changshu Medicine Examination Institute, Changshu, Jiangsu 215500, PR China

**Keywords:** Norovirus, Receptor, Vaccine, Antiviral

## Abstract

*Norovirus*, belonging to the *Caliciviridae* family, is a non-enveloped, positive-sense single-stranded RNA virus. It is widely acknowledged as a significant etiological agent responsible for non-bacterial acute gastroenteritis and considered a major cause thereof. *Norovirus* is primarily tranmitted via fecal-oral route, but can also be transmitted via airborne routes. Clinical manifestations often include symptoms associated with acute gastroenteritis, like nausea, vomiting, watery diarrhea, stomach cramps, and others. Due to the specific pathogenic mechanism of the virus, and genomic diversity, there are currently no preventive vaccines or effective antiviral drugs available for treating norovirus-induced acute gastroenteritis infections. The management of such infections mainly relies on oral rehydration therapy while prevention necessitates adherence to personal hygiene measures.

The present paper discusses the nature, transmission route, clinical manifestations, immune response mechanism, and vaccine research of *Norovirus*. The objective of this review manuscript is to systematically gather, analyze, and summarize recent research and investigations on norovirus in order to enhance our understanding of its characteristics and pathogenesis. This not only facilitates subsequent researchers in acquiring a more expedited and comprehensive grasp of the existing knowledge about norovirus but also provides clearer directions and goals for future studies.

## Highlights

• A comprehensive review of the basic structure, pathogenic characteristics, clinical manifestations, immune response mechanisms, and vaccine research of norovirus.
• A detailed description of the transmission routes, genetic classification, genotypes, and serotypes of norovirus, as well as its global epidemic situation.
• Discussion of the prevention and treatment of norovirus infection, including personal hygiene, vaccination, and antiviral drugs.
• Provides the latest progress in norovirus research, providing guidance for subsequent research.

## 1 Introduction of *norovirus*

### 1.1 Basic structure and pathologic profile of norovirus

Belonging to the Caliciviridae family, norovirus is a non-enveloped, positive-sense single-stranded RNA virus (ssRNA(+) virus) that can be classified into 10 genogroups (Chhabra et al., 2019; Li et al., 2023). The norovirus was first identified in 1968 during an outbreak of acute gastroenteritis in Norwalk, Ohio, USA, where it was isolated from the feces of affected patients (Kapikian et al., 1972). Since then, this virus has been consistently detected in the fecal samples from patients with gastroenteritis worldwide. Human norovirus is widely recognized as a significant etiological agent of acute gastroenteritis (Lu et al., 2023; Fang et al., 2021), accounting for approximately 20 % of cases in children under the age of five (Hall et al., 2013). Some genogroups of norovirus infect animals like dogs,pigs and mice, while others infect humans (Chhabra et al., 2019). The primary mode of transmission is through the fecal-oral route (Rexin et al., 2024; Dolin et al., 1971), and involves ingesting contaminated food or water and person-to-person contact (Mao et al., 2023; Phattanawiboon et al., 2020; Verhoef et al., 2015; Ji et al., 2020). However, there is also evidence suggesting the norovirus can be transmitted by the airborne route (Alsved et al., 2020). In recent years, there has been a steady increase in the incidence of acute gastroenteritis associated with norovirus. Clinical manifestations often include symptoms such as nausea, vomiting, watery diarrhea, gastrointestinal spasms, etc., occasionally accompanied by fever, chills, headache and general malaise (Ku et al., 2014). Additionally, combined with the research in recent years has indicated that norovirus can induce benign seizures in conjunction with acute gastroenteritis, suggesting a potential association with the nervous system (Moreno-Valladares et al., 2022). From a clinical perspective, it has been observed that individuals who remain asymptomatic following infection demonstrate comparable transmissibility to symptomatic patients (Phattanawiboon et al., 2020). This poses a significant challenge for accurate clinical identification of norovirus.

### 1.2 The patterns of viral invasion in the host

**Fig. 1:**
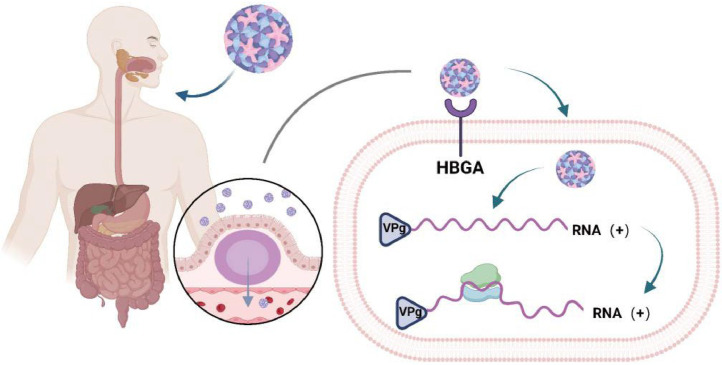
Hypothetical model of *norovirus* infection pathogenesis based on findings from patients infected with norovirus.

The primary mode of norovirus invasion into the body is through infection of macrophages and dendritic cells in the gastrointestinal tract (Karst and Tibbetts, 2016; Donaldson et al., 2008). Upon infecting humans, these viruses exhibit a preference for targeting the gastrointestinal tract, Norovirus can use M cells across the intestinal epithelial barrier and enterocytes are able to collect material from the intestinal lumen and transfer them to potential immune cells (Karst and Tibbetts, 2016). It is worth noting that most studies investigating this mechanism have primarily utilized mice or other caliciviruses due to challenges associated with successfully culturing norovirus in human cells (Thorne and Goodfellow, 2014) The invasion of norovirus is mainly divided into five steps, which are attachment, receptor binding, endocytosis, uncoating, and translation (Marsh and Helenius, 2006). According to limited reports, the attachment of murine norovirus to the HBGAs structure on the cell membrane is facilitated by various factors, including bile acids, sialic acids, and cations (Taube et al., 2009), and then norovirus binds to CD300lf (Kolawole et al., 2014). Ceramide plays an important role in the binding process. In contrast to most viruses that rely on pH value for endocytosis (Shivanna et al., 2014), norovirus mainly relies on cholesterol and dynein for endocytosis (Gerondopoulos et al., 2010). Lacking the 5 'cap structure (Emmott et al., 2017), norovirus RNA relies on the VPg protein to bind to initiation factors to initiate the translation process as showed in Fig. 1.

### 1.3 The viral subtype

The gene classification of norovirus is currently categorized into ten distinct genogroup (*Norovirus GI* to *Norovirus GVX*) and two unassigned gene groups (Chhabra et al., 2019; Degiuseppe et al., 2020). Through the comparison of *norovirus* gene sequences, researchers have further classified these gene groups into 49 distinct capsid genotypes (Cao et al., 2022) and 60 unique p types (Chen et al., 2023; Wang et al., 2021), significantly enhancing our comprehension of norovirus. The *GI* and *GII* noroviruses deserve special attention due to their pandemic (Cao et al., 2022; Chen et al., 2023; Zhou et al., 2020).

**Fig. 2:**
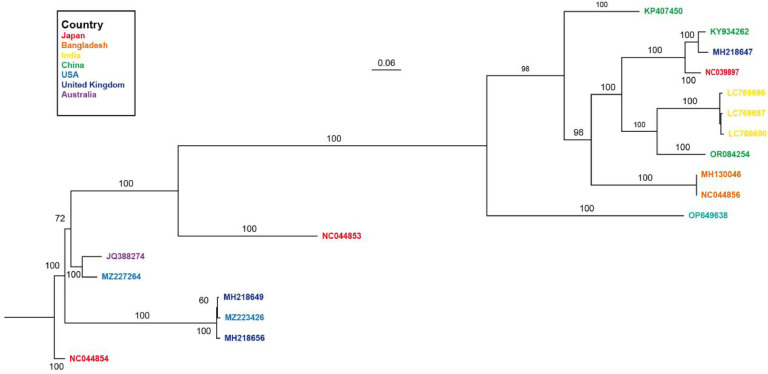
Gene evolution tree in complete genome sequences of 18 global norovirus subtypes GI. The tree represents the evolutionary history of the gene family through the transmission of norovirus, as well as the phylogenetic relationships between various norovirus global epidemic strains in different regions and countries (see color legend). "0.006″ represents the scale of branch lengths, which are used to measure the distance between ancestors and descendants. Other numbers of internal nodes, also known as the support value, is used to evaluate how reliable a branch structure is. The branch is supported by more evidence the higher the value.

The complete genome sequences of serotypes *GI* and *GII*, which possess the highest global epidemic potential, were obtained from GenBank and utilized as reference strains for phylogenetic analysis. The sequence information can be found in Supplementary Materials, Table S.1. We utilized the Align By Muscle functionality in MEGA 10.1.8 (Kakakhel et al., 2022; Pazdiora et al., 2021) to perform a comparative analysis of the downloaded sequences, and subsequently construct a Bayesian inference tree for the entire gene using MrBayes v3.2.7 (Ronquist et al., 2012; Portela et al., 2021). The obtained results unveiled numerous variants of norovirus strain *GI,* as showed in Fig. 2.

**Fig. 3:**
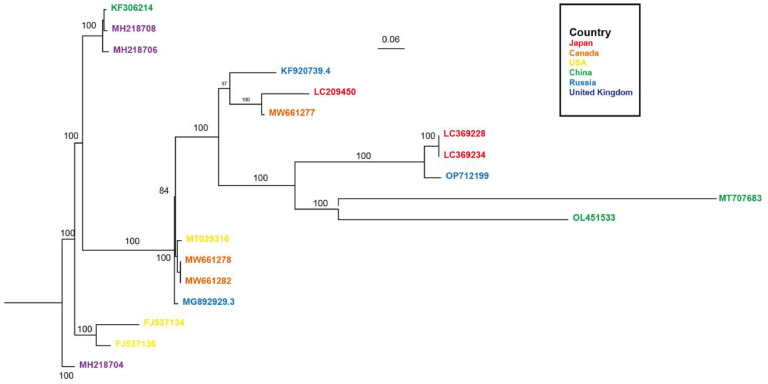
Gene evolution tree in complete genome sequences of 18 global norovirus subtypes *GII*. The tree was generated as the methods of Fig. 2.

The numerous variants of norovirus strain *GII was depicted in* Fig. 3. The ongoing variability in strains poses challenges for the development of a universal vaccine that can effectively neutralize a wide range of strains and subgenotypes. Consequently, individuals who have received full vaccination may still be susceptible to infection, particularly if the vaccine's efficacy is compromised. Therefore, there is an urgent need for novel antiviral drugs alongside advancements in vaccine research.

### 1.4 Public health strategies to prevent *norovirus* infection

In the research and development of vaccines, numerous challenges exist in developing effective solutions for norovirus, including the intricate nature of human natural immune response to norovirus (Donaldson et al., 2008; Ford-Siltz et al., 2021), the genetic diversity within the viral norovirus genome, the absence of a reliable viral in vitro culture system (Ettayebi et al., 2016), and limited animal models available for vaccine testing. Consequently, an approved norovirus vaccine has yet to be established. Fortunately, several studies have reported that zebrafish can serve as a novel HuNov model, offering valuable insights for the investigation of norovirus vaccines (Zhang et al., 2021). On the other hand, for acute gastroenteritis caused by norovirus, the current primary treatment is oral rehydration therapy. This included a rehydration phase involving the intake of oral rehydration electrolytes and water, as well as a maintenance stage treatment that involved consuming electrolytes, water, and appropriate food (Bonvanie et al., 2021). Research has demonstrated that ondansetron can be used to alleviate symptoms in children experiencing vomiting (Hartman et al., 2019). Meanwhile, the public health care program should be further strengthened to effectively prevent viral infections. Additionally, measures should be implemented to enhance daily life and minimize the risk of viral infection. These measures primarily rely on consistent adherence to hand hygiene practices, meticulous attention to sanitizing environmental surfaces, and minimizing direct contact with infectious sources.

## 2 Receptors and distribution

The tissue and cell tropism of viruses is determined by numerous factors, with host cell surface receptors playing a pivotal role in this process. By investigating the surface receptors of noroviruses, we can gain insights into their invasion mechanisms and characteristics within the human body. According to the previous studies, CD300 is identified as the murine norovirus receptor (Graziano et al., 2020), which is a membrane protein containing immunoglobulin (Ig) domains. In contrast, human norovirus receptors can be mainly classified into two categories: one being carbohydrate receptors represented by histological blood group antigens (HBGAs) (Harrington et al., 2002), and the other being immune receptors represented by Toll-like receptor (TLR) (Finberg et al., 2007). The expression site and mode of action of the human norovirus receptors on the host are summarized in Table 1.

**Table 1:** The expression site and mode of action of the human norovirus receptors on the host.

Name	Site of expression	Function	Virus binding mode	References
HBGAs	Respiratory tract, digestive tract, urogenital mucous epithelium; saliva and other biological fluids; red blood cell	Promoting the interaction between the viral P structure and the binding pocket of HBGAs.	Reconstructing HBGA binding site (HBS) through α-Fuc interaction with HBGAs and 444 mutation of VP1 to tyrosine, HBS changes through 382 mutation of VP1 and the molecular pattern of oligosaccharide binding	(Jin et al., 2015; Kher et al., 2023; Kimura-Someya et al., 2022; Yu et al., 2023)
TLR2	Plasma, endosome, within the lysosome membrane	Infect, promote inflammation	Dose-dependent approach of VLPs	(SantisoBellón et al., 2022)
TLR5		Induce mucosal and systemic IgA and IgG		(Enosi Tuipulotu et al., 2018; Hjelm et al., 2014)

Carbohydrate binding is a prevalent mechanism employed by most viruses and microorganisms to adhere to the host cell (Zheng et al., 2020). Among these, HBGAs represent a family of carbohydrate structures that utilize the carbohydrate binding mode as their primary receptor for norovirus attachment to the host cell. The catalysis of HBGAs is facilitated by glycosyltransferases encoded by three gene families (ABO, secretor, and Lewis) (Kimura-Someya et al., 2022; Peña-Gil et al., 2021; Sharma et al., 2020; Ibaraki et al., 2023), which sequentially add monosaccharides to the precursors. HBGAs are predominantly present in the mucosal epithelium of the respiratory, alimentary, and urogenital tracts, as well as in the form of free oligosaccharides like saliva (Nordgren and Svensson, 2019; Tenge et al., 2021; Villabruna et al., 2021; Cong et al., 2023). The FUT3 enzyme facilitates the conversion of the H type 1 antigen into Lewis b (Kimura-Someya et al., 2022; Auger et al., 2023). The A and B enzymes encoded by the ABO (Villabruna et al., 2021; Marionneau et al., 2001; Liao et al., 2020) gene can enzymatically add acetylgalactosamine or galactose to the H antigen. According to the different subtypes of norovirus, strains can bind to HBGAs in various ways. The example of *GII* strains here is taken to illustrate their primary interaction with HBGAs through α-Fuc residues (Frenck et al., 2012). For example, the *GII.17* strain enhanced its HBGA binding capacity by modifyingits HBGA binding site (HBS) through a mutation at position 444 in VP1 (Qian et al., 2019), substituting it with tyrosine (Yi et al., 2021) . Additionally, the HBS of the *GII.2* BJSMQ outbreak strain underwent a mutation at position 382 in VP1 (Cong et al., 2023; Ao et al., 2018), thereby increasing its stability and binding capacity. On the other hand, the ability of viruses with low HBGA binding capacity to trigger outbreaks is attributed to alterations in the molecular patterns of oligosaccharide binding (Auger et al., 2023). Relevant studies have demonstrated that norovirus employed its own P structure for HBGAs binding (Sun et al., 2021) through a process referred to as binding bags. Both cellular and non-cellular host molecules enhanced the binding of norovirus. For instance, bile salts could induce moderate structural alterations in the P structural domain to facilitate norovirus binding (Nelson et al., 2018; Graziano et al., 2019), while ceramides might modify the conformation of antibody epitopes on cells to promote virus entry (Almand et al., 2017; Orchard et al., 2018; Creutznacher et al., 2023).

The immunoreaction is a crucial antiviral defence response system that plays a pivotal role in the recognition and elimination of intracellular pathogens. Toll like receptors (TLRs), expressed in various innate immune cells and localized within the plasma, endosome, and lysosome membranes, represents a family of transmembrane receptors. The virus-like particles (VLPs) of the norovirus protein coat exhibit a dose-dependent binding to TLR2 and TLR5 (Ponterio et al., 2019) receptors on host cell surfaces, thereby eliciting an immune-mediated response. The previous studies have reported that the TLR2 pathway was implicated in viral host infection and inflammation (Cheng et al., 2017) promotion, whereas the TLR5 pathway significantly elicited mucosal and systemic IgA and IgG responses (Ibaraki et al., 2023; Verma et al., 2016; Kirby et al., 2020).

In general, norovirus can enter host cells through various receptors, and the expression of these receptors varies across different tissues (Liang et al., 2021). Currently, receptor studies on norovirus primarily focus on HBGAs, which have been documented in reports dating back to December 2003 (Huang et al., 2003). Follow-up studies have revealed that multiple attachment factors played a pivotal role in facilitating the binding of norovirus to HBGAs, as well as immune receptors such as TRLs. The diverse array of receptors gradually unraveled the invasive mechanisms and characteristics of norovirus. However, there are still certain mechanisms and receptors that remain incompletely understood. Given the continuous evolution of norovirus and expansion of its genetic pool, researchers need to explore an even broader range of receptor subtypes.

## 3 Clinical observation

The most prevalent symptoms associated with norovirus infection include emesis (Alsedà et al., 2023) and diarrhea (Lin et al., 2010), occasionally accompanied by nausea (Sun et al., 2022), abdominal discomfort, cramping (Alsedà et al., 2023), anorexia (Hellysaz et al., 2023), malaise, and low-grade pyrexia (Fang et al., 2022). Common complications may encompass benign seizures (Chen et al., 2009), febrile seizures (Chan et al., 2011), convulsions (Shi et al., 2023), necrotizing enterocolitis (Turcios-Ruiz et al., 2008) as well as exacerbation of inflammatory bowel disease (Khan et al., 2009). Detection of respiratory secretions from children presenting with respiratory symptoms revealed that norovirus may also be implicated in respiratory diseases (Esposito et al., 2014). Additionally, there have been reported cases where mild meningoencephalitis (Kimura et al., 2010) can be attributed to norovirus infection based on clinical manifestations.

The main symptoms associated with norovirus infection exhibited variability among individuals, with a higher prevalence observed in infants, the elderly, and immunocompromised individuals (Lopman et al., 2004). Studies have indicated that young children exhibit more severe symptoms, with the establishment of protective immunity to prevent re-infection typically occurring before the age of 2 years (Sai et al., 2013). The primary manifestations of diarrhea in infants under 1 year old include watery diarrhea (Shang et al., 2016), which can lead to dehydration, electrolyte imbalance, convulsions, and potentially fatal outcomes in severe cases.

In addition, the patients with norovirus gastroenteritis accompanied by convulsions also experience transient vomiting (lasting for approximately 2 days) in the absence of fever (Chen et al., 2019). For patients with immunocompetent norovirus gastroenteritis, the incubation period of infection is typically short, usually ranging from 24 to 48 h (Lively et al., 2018). This condition is characterized by symptoms such as vomiting, nausea, abdominal cramps, and diarrhea, which generally resolve within a span of 72 h (Lewis et al., 2023).

Furthermore, the various strains of the virus would exhibit distinct clinical manifestations. According to previous studies, the *GII* genotype is globally prevalent in human norovirus infections, while the *GI* genotype is only observed in a limited number of cases (Fardah Athiyyah et al., 2019; Sun et al., 2023). Considering seasonal variations, the prevalence of *GI* genotypes is primarily observed during spring, whereas *GII* genotypes are predominant during autumn (Cao et al., 2021; Chan et al., 2018; Chiu et al., 2022). Compared to other genotypes, norovirus *GII* infection is associated with a higher incidence of severe diarrhea and vomiting (Melhem et al., 2016). Additionally, a greater proportion of patients infected with genotype *GI* experienced symptoms such as nausea, diarrhea lasting less than 3 days, and increased bowel sounds (Xue et al., 2015).

The comprehension of dominant strains and their correlation with clinical manifestations will facilitate the enhancement of clinical diagnosis accuracy and the development of evidence-based prevention and treatment strategies.

## 4 The structure of norovirus and the host immune response against its pathogenesis

The norovirus genome typically consisted of three open reading frames (ORFs) (Dan et al., 2020), wherein ORF1 was encoded six non-structural (NS) proteins (Belliot et al., 2003), namely p48, nucleoside triphosphatase (NTPase), p22, VPg, protease (Pro), and RNA-dependent RNA polymerase (RdRp). The major structural protein of the viral capsid VP1 was encoded by ORF2, while the minor structural protein of the capsid VP2 was encoded by ORF3 (Lin et al., 2014; Liu et al., 2019).

Upon binding to the cellular receptor via the P domain of the VP1 protein, the virus internalized and unshelled, releasing positive RNA (+RNA) in the cytoplasm (Tan et al., 2004). Replication was completed through the coordinated action of Pol, VPg, and NTPase proteins (Li et al., 2018). Subsequently, the VPg protein binds to the 5′ end of the RNA, recruited initiation factors, and participates in initiating viral RNA translation for producing VP1 and VP2 proteins (Olspert et al., 2016).

**Fig. 4:**
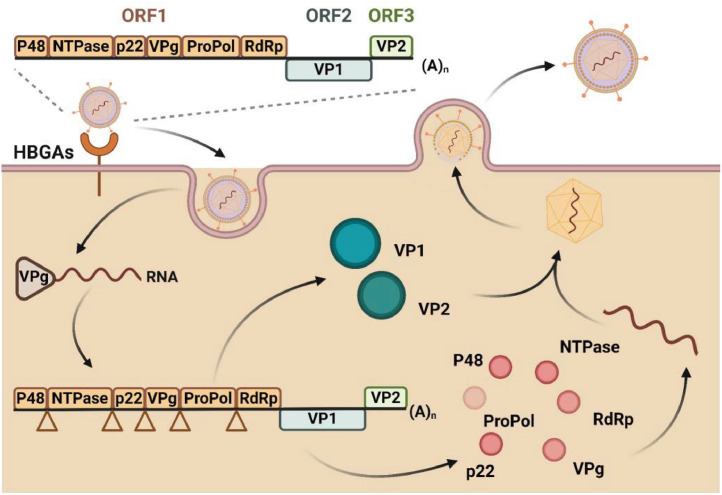
The significance of viral structure in the processes of replication and infection.

Following successful translation, the majority of proteins encoded by norovirus ORF1 undergo proteolytic processing mediated by the viral-encoded protease (Pro/NS6), resulting in their cleavage into distinct entities: p48 (NS1/2), NTPase (NS3), p22 (NS4), VPg, Pro, and RdRp (May et al., 2014). These individual proteins possessed specific functionalities crucial for replication and infection processes. The process of norovirus entry and replication through its various structural elements was illustrated in Fig. 4.

And the comprehensive information regarding the ORFs and their encoded proteins of norovirus, which played a pivotal role in both replication and infection processes, was summarized in Table 2.

**Table 2:** The genomic architecture of norovirus plays a crucial role in facilitating its molecular functions during replication and infection processes.

ORFs	Proteins encoded by ORFs	Nomenclature	Molecular Function	References
ORF1	NS1/2	p48 N-terminal	The participation in the formation of viral replication complex, induction of aberrant cellular calcium signaling, promotion of viral replication, and evasion of IFN-λ-mediated antiviral immunity are observed.	(Hung et al., 2023; Lee et al., 2017)
	NS3	NTPase	The protein possesses NTP-dependent RNA helicase activity as well as NTP-independent RNA chaperone activity, enabling it to unwind and disrupt the structural integrity of RNA molecules.	(Li et al., 2018)
	NS4	p22	The induction of vesicle structures can potentially be associated with the formation of replication vesicles induced by norovirus.	(Doerflinger et al., 2017)
	NS5	VPg	The binding of translation initiation factor elF3 participates in RNA translation and interacts with the protein cap-binding complex elF4F; It also has been shown to induce cell cycle arrest in the G0/G1 phase.	(Daughenbaugh et al., 2006; McSweeney et al., 2019; McSweeney et al., 2021)
	NS6	Pro 3CLpro NS6pro	The major polymerase responsible for nucleotideylation.	(Medvedev et al., 2017)
	NS7	RdRp NS7pro	RdRp could direct viral replication. RdRp protein may have affected the evolution of the VP1 gene/VP1 protein.	(Takahashi et al., 2023; Obaid et al., 2023)
ORF2	VP1	Viral protein 1	The major structural protein of the norovirus capsid binds to human blood group antigens (HBGAs) through its p structure.	(Strong et al., 2012)
ORF3	VP2	Viral protein 2	A secondary structural protein responsible for the nuclear transport of VP1.	(Liu et al., 2019)

The pathogenesis of norovirus is characterized by intricate host-virus interactions, wherein numerous factors play pivotal roles, including histiocyte tropism, host immune response, and bacterial microorganisms. The cytotaxis of norovirus primarily targets gut-associated lymphoid tissue (GALT) immune cells in the distal ileum, encompassing Macrophages(Mφ), Dendritic cells(DCs), B cells, and T cells; alongside infrequent cell populations within the follicle-associated epithelium (FAE) (Grau et al., 2017).

Some chemokines/cytokines have the function to suppress the virus replication. During viral infection, both type I and type II interferons can suppress the replication of replicon expression by inducing innate immune mediators, as well as impede the interaction between VPg and translation initiation factors, thereby inhibiting the translation of non-structural proteins (Neil et al., 2019; Strohmeier et al., 2023). The action of IL-4 on cluster cells enhances the immune response (Wilen et al., 2018). Additionally, the B and T cells of the adaptive immune system (Hsu et al., 2018; Malm et al., 2016) play a crucial role in effectively eliminating intestinal and enteric lymph node viruses. The outer membrane vesicles (OMV) (Grau et al., 2017; Mosby et al., 2022; Mosby et al., 2023) play a crucial role in facilitating communication between symbiotic bacteria and their hosts. Following norovirus infection, intestinal symbiotic bacteria exhibit an enhanced production of bacterial outer membrane vesicles (BEV) (Bhar et al., 2022), which contribute to the regulation of immune responses.

## 5 The administration of pharmaceuticals and immunizations

In drug research, there are three main categories of antiviral drugs based on their mechanisms: the first category aims to prevent viruses from attaching to host cell surfaces. For example, HBGAs have been identified as important factors promoting virus adhesion and entry. Research has shown that certain caramel carbohydrates, such as citric acid salt (Ali et al., 2016), can effectively inhibit viral activity and hold great potential in antiviral drug development. The second category involves virus protease inhibitors which target the catalytic decomposition of replication-related proteins by viral proteases (Rocha-Pereira et al., 2014). Rupintrivir is a notable antiviral drug within this category, primarily used for treating human rhinovirus but also showing efficacy against other picornaviruses, coronaviruses and caliciviruses (Kim et al., 2012). The third category consists of RdRp inhibitors, with nucleoside analogues being the most representative examples. These compounds block nucleotide binding and thereby hinder viral invasion. Currently, purine nucleoside CM521 is the only approved drug for norovirus infection treatment (Santos-Ferreira et al., 2021); followed by 20-C-methylcytidine (2CMC), a popular cytidine analogue that inhibits virus polymerase activity and translation; additionally, adenosine analogues like 7-deaza-20-C-methyladenosine (7DMA)and NITD008 exhibit similar properties (Van Dycke et al., 2018).

Firstly, natural products derived from plants such as black raspberry seed, laminaceous algae polysaccharide, aged green tea, grass seed asarum extract, and grape seed extract (GSE) have been investigated. GSE has been confirmed to contain over 83 % proanthocyanidins which primarily function in virus aggregation and significantly inhibit viral invasion and replication (Kudkyal et al., 2022; Oh et al., 2022). Similarly, the rhizomes of Acorus gramineus(AGR) extract along with ɑ-asarone and β-asarone from grass seed asarum exhibited notable antiviral effects by preventing viral binding to P structure (Kim et al., 2022). Similar findings were observed with pomegranate peel extract (PPE) (Živković et al., 2021). Secondly, synthetic compounds like Dasabuvir (DSB), originally developed for hepatitis C treatment (Hayashi et al., 2021) but incidentally discovered to impede norovirus development within the body (Hayashi et al., 2021; Netzler et al., 2019) represent another group of potential therapeutics.

In terms of vaccines, the development of an effective norovirus vaccine is crucial for reducing norovirus-related morbidity and mortality. According to recent researches, norovirus vaccines can be categorized into two groups: VLP vaccines and P particle vaccines. Within the VLP vaccine category, there are further classifications based on the composition of the norovirus capsid protein VP1 (VLP-based) and the expression of VP1 using recombinant adenoviruses (recombinant adenovirus-based) (Verardi et al., 2020). As depicted in Table 3, existing norovirus vaccines possess distinct characteristics, advantages, and disadvantages due to their different types and sources.

**Table 3:** Overview of existing norovirus vaccines.

Vaccine	Immunization route	Clinical Progress	Advantage	Shortage	Reference
VLP vaccine	Nasal entry	The clinical stage has been reached with corresponding Phase II b clinical trials underway.	The research on VLP vaccines is supported by extensive experience and experimental data.	The presence of associated adverse effects necessitates further refinement.	(Zhang et al., 2021; Chen et al., 2020)
Recombinant viral vaccine	Oral	The expression of the target has been demonstrated in murine models and advanced to phase Ⅰ B clinical trials.	The reduction in the number of injections necessitates a single dose. The immunological response is enhanced.	The development, availability, and effectiveness may be constrained by biosafety concerns and preexisting host immunity.	(López et al., 2023; Panasiuk et al., 2021; Hallowell et al., 2019; Hanisch, 2022; Han et al., 2021)
P-particle vaccine	NA	The preclinical studies have been successfully concluded.	The compound has shown improved immune responses, similar to virus-like particles (VLPs), in terms of immunogenicity and protective efficacy.	Effectiveness and duration need to be enhanced.	(Hallowell et al., 2019; Yang et al., 2019)

In conclusion, the development of norovirus vaccines and drugs has progressed rapidly. Concurrently, more clinical trials and animal experiments are underway to further promote their advancement. Vaccine research will provide novel ideas and directions for drug development, while drug innovation will explore additional possibilities for vaccine improvement.

## 6 Future outlook

Norovirus accounts for approximately 20 % of the global burden of acute gastroenteritis (Atmar et al., 2018; Cortes-Penfield et al., 2017). According to available literature, norovirus is responsible for an estimated 698.8 million cases of illness and 218,800 deaths annually across all age groups (Bartsch et al., 2016). However, limited research content and findings hinder a comprehensive understanding of norovirus.

According to the correlated characteristics of norovirus, future research on antiviral drugs and vaccines could focus on inhibiting virus invasion or blocking virus binding with receptors on the surface of norovirus, such as HBGAs and TLR. Additionally, targeting specific proteins expressed by the genome of norovirus can inhibit viral replication, transcription, and translation to facilitate the development of antiviral drugs and vaccines. Furthermore, plant-derived substances or synthesized drugs can be utilized to impede viral pathogenesis for treatment and prevention purposes. Currently, numerous studies have been conducted on drugs and vaccines with some pharmaceutical companies already progressing related products through clinical experiments. It is anticipated that more effective drugs will be developed in the future leading to improved research and treatment outcomes for norovirus.

## Abbreviation

**Table:** 

Fill title	Abbreviation	Fill title	Abbreviation
Norovirus	Nov	Histoblood group antigen	HBGAs
Immune globulin	Ig	Toll-like receptors	TLR
Hisblood group antigen binding site	HBS	Fucosyltransferase 2	FUT2
Virus-like particles	VLPs	Open reading frame	ORF
Non-structural protein	NSP	Nucleoside triphosphatase	NTPase
Protease	Pro	RNA-dependent RNA polymerase	RdRp
Gut-associated lymphoid tissue	GALT	Macrophages	Mφ
Dendritic cells	DCs	Follicle-associated epithelium	FAE
Outer membrane vesicles	OMV	Bacterial outer membrane vesicles	BEV
Grape seed extract	GSE	The rhizomes of Acorus gramineus	AGR
Pomegranate peel extract	PPE	Dasabuvir	DSB

## Funding declaration

This work was supported financially by the 10.13039/501100001809National Natural Science Foundation of China (82101630) to Yuwei Liu.

## CRediT authorship contribution statement

**JunLi Chen:** Project administration. **ZhengChao Cheng:** Project administration. **Jing Chen:** Project administration. **Lingling Qian:** Project administration, Supervision. **Haoran Wang:** Supervision, Funding acquisition. **YuWei Liu:** Supervision, Funding acquisition, Conceptualization.

## Declaration of competing interest

The authors declare no conflict of interest.

## Data Availability

No data was used for the research described in the article.

## Appendix Supplementary materials

Image, application 1
