# Supplementary material for: Advances in human norovirus research: Vaccines, genotype distribution and antiviral strategies
Source: Virus Res. 2024 Oct 23;350:199486. doi: 10.1016/j.virusres.2024.199486 (PMC11539660; doi:10.1016/j.virusres.2024.199486)
Supplement: Supplementary file 1 [file mmc1.docx]

**Supplementary**

| GenBank Nos. | Description |
| --- | --- |
| JQ388274.1 | \|Norovirus Hu/GI.6/Kingston/ACT160D/2010/AU, complete genome |
| KP407450.1 | \|Norovirus GI strain Hu/CHN/2008/GI.P8_GI.8/Huzhou/N10, complete genome |
| KY934262.1 | \|Norovirus GI isolate 0304-19, complete genome |
| LC769686.1 | \|Norovirus GI Hu/IN/2019/GI.3[P13]/NICED-BCH-10876, nearly complete genome |
| LC769687.1 | \|Norovirus GI Hu/IN/2019/GI.3[P13]/NICED-BCH-10892, nearly complete genome |
| LC769690.1 | \|Norovirus GI Hu/IN/2019/GI.3[P13]/NICED-BCH-11157, nearly complete genome |
| MH130046.1 | \|Norovirus GI strain Hu/BD/2011/GI.Pg-GI.7/Dhaka1882, complete genome |
| MH218647.1 | \|Norovirus GI isolate NORO_179_03_08_2015, complete genome |
| MH218649.1 | \|Norovirus GI isolate NORO_181_09_09_2015, complete genome |
| MH218656.1 | \|Norovirus GI isolate NORO_188_01_10_2015, complete genome |
| MZ223426.1 | \|Norovirus GI isolate Hu/US/2014/GI.2[P2]/CA-RGDS-1105, complete genome |
| MZ227264.1 | \|Norovirus GI isolate Hu/US/2016/GI.6[P11]/CA-RGDS-1110, complete genome |
| NC_039897.1 | \|Norovirus GI/Hu/JP/2007/GI.P3_GI.3/Shimizu/KK2866, complete genome |
| NC_044853.1 | \|Norovirus GI strain Hu/JP/1998/GI.6[PNA4]/No20-Saitama-98-17, complete genome |
| NC_044854.1 | \|Norovirus GI strain Hu/JP/2000/GI.6[PNA1]/WUG1, complete genome |
| NC_044856.1 | \|Norovirus GI strain Hu/BD/2011/GI.7[PNA2]/Dhaka1882, complete genome |
| OP649638.1 | \|Norovirus GI isolate Hu/US/2021/GI.9[P9]/CA-RGDS-1150, complete genome |
| OR084254.1 | \|Norovirus GI isolate 2017-310, complete genome |

TableS.1 Sequence information of *Norovirus GI* for the phylogenetic analysis

| GenBank Nos. | Description |
| --- | --- |
| FJ537134.1 | \|Norovirus Hu/GII.4/CHDC5191/1974/US, complete genome |
| FJ537136.1 | \|Norovirus Hu/GII.4/CHDC3967/1988/US, complete genome |
| KF306214.1 | \|Norovirus Hu/GII.4/Jingzhou/2013403/CHN, complete genome |
| KF920739.4 | \|Norovirus GII strain Hu/GII.P16-GII.16/RUS/Novosibirsk/Nsk-N4740/2012, complete genome |
| LC209450.1 | \|Norovirus Hu/GII/JP/2014/GII.P16_GII.2/Osaka-225 RNA, nearly complete genome |
| LC369228.1 | \|Norovirus GII NoV/Hu/JP/2014/GII.P17-GII.17/MI-77 genomic RNA, nearly complete genome |
| LC369234.1 | \|Norovirus GII NoV/Hu/JP/2015/GII.P17-GII.17/MI-85 genomic RNA, nearly complete genome |
| MG892929.3 | \|Norovirus GII strain Hu/GII.P16-GII.4/RUS/Novosibirsk/NS17-A869/2017, complete genome |
| MH218704.1 | \|Norovirus GII isolate NORO_46_24_08_2014, complete genome |
| MH218706.1 | \|Norovirus GII isolate NORO_48-1_01_07_2014, complete genome |
| MH218708.1 | \|Norovirus GII isolate NORO_50_30_07_2014, complete genome |
| MT029316.1 | \|Norovirus GII isolate Hu/US/2018/GII.4 Sydney[P16]/Helena0207, complete genome |
| MT707683.1 | \|Norovirus GII isolate KMN1 nonstructural polyprotein (ORF1) gene, partial cds; and VP1 (ORF2) and VP2 (ORF3) genes, complete cds |
| MW661277.1 | \|Norovirus GII isolate BMH19-125 nonstructural polyprotein (ORF1), VP1 (ORF2), and VP2 (ORF3) genes, complete cds |
| MW661278.1 | \|Norovirus GII isolate BMH19-127, complete genome |
| MW661282.1 | \|Norovirus GII isolate BMH19-132, complete genome |
| OL451533.1 | \|Norovirus GII isolate NS21078, complete genome |
| OP712199.1 | \|Norovirus GII isolate GII/Hu/RU/2022/GII.17[P17]/Nizhny_Novgorod755, complete genome |

TableS.2 Sequence information of *Norovirus GII* for the phylogenetic analysis
